# Errata

**DOI:** 10.1289/ehp.121-a455

**Published:** 2012-12-01

Joubert et al. [Environ Health Perspect 120:1425–1431 (2012)] have reported errors in their paper, “450K Epigenome-Wide Scan Identifies Differential DNA Methylation in Newborns Related to Maternal Smoking During Pregnancy.” In Figure 2, the gene name for the last CpG listed, cg12477880, should have been RUNX1 instead of CYP1A1. In Table 3, the percent differences in median methylation (smokers – nonsmokers) were incorrect for two CpGs; the correct values are –1.2 (MoBa) and –1.1 (NEST) for TTC7B cg18655025, and –1.8 (MoBa) and –0.3 (NEST) for HLA-DPB2 cg11715943. These values are presented correctly in Figure 2. EHP and the authors regret the errors.

Li et al. have provided an update for their paper “Differential Estrogenic Actions of Endocrine-Disrupting Chemicals Bisphenol A, Bisphenol AF, and Zearalenone through Estrogen Receptor α and β in Vitro” [Environ Health Perspect 120:1029–1035 (2012)]. Recent analysis indicates that the mouse ERβ expression plasmid used in their study had a mutation of 310 glutamic acid (E) to glycine (G).

